# Supplementary material for: BRAF V600E Mutation in Ameloblastoma: A Systematic Review and Meta-Analysis
Source: Cancers (Basel). 2022 Nov 14;14(22):5593. doi: 10.3390/cancers14225593 (PMC9688909; doi:10.3390/cancers14225593)
Supplement: Supplementary file 1 [file cancers-14-05593-s001.zip › cancers-1902198-supplementary.pdf]

Supplementary

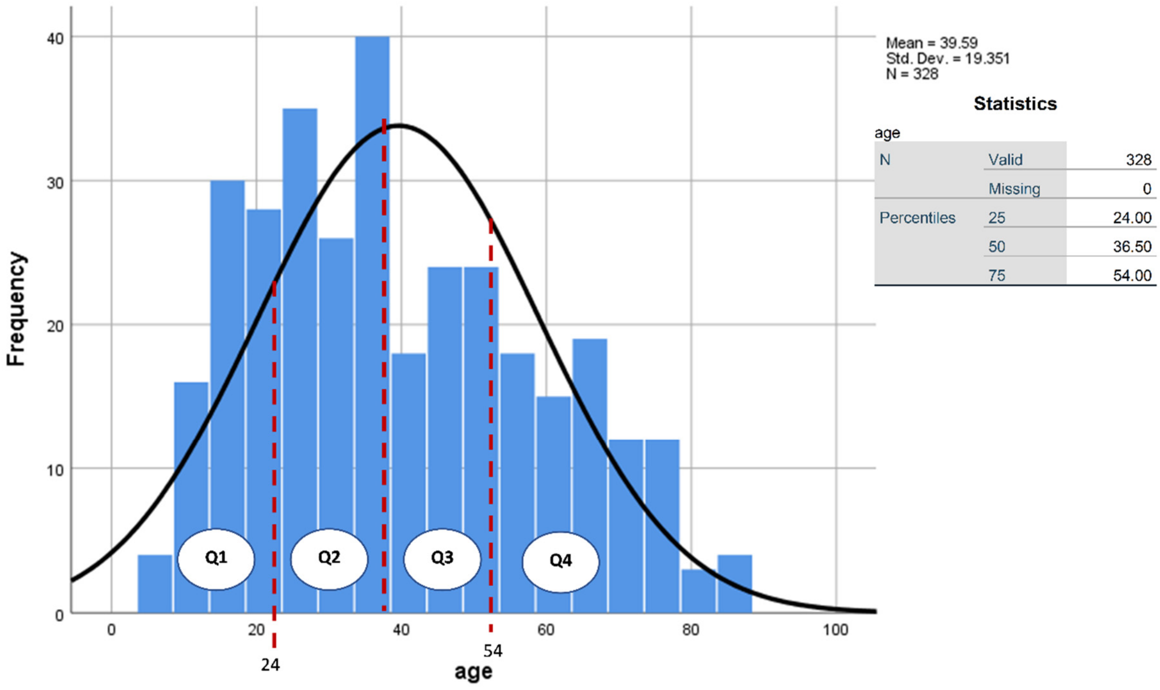

Figure S1: Histogram of age group distribution and quartiles. Q1, quartile 1 (age below 24 years old); Q2, quartile 2 (age between 24 and 37 years old); Q3, quartile 3 (age between 37 and 54 years old); Q4, quartile 4 (age above 54 years old).
